# Therapeutic Potential of Mesenchymal Stem Cells in Regenerative Medicine

**DOI:** 10.1155/2013/496218

**Published:** 2013-03-19

**Authors:** Devang M. Patel, Jainy Shah, Anand S. Srivastava

**Affiliations:** ^1^GIOSTAR/Saviour Hospital, Near Lakhudi Talav, Stadium Road, Navrangpura, Ahmedabad, Gujarat 380014, India; ^2^Global Institute of Stem Cell Therapy and Research, 4370 La Jolla Village Drive San, Diego, CA 92122, USA

## Abstract

Mesenchymal stem cells (MSCs) are stromal cells that have the ability to self-renew and also exhibit multilineage differentiation into both mesenchymal and nonmesenchymal lineages. The intrinsic properties of these cells make them an attractive candidate for clinical applications. MSCs are of keen interest because they can be isolated from a small aspirate of bone marrow or adipose tissues and can be easily expanded *in vitro*. Moreover, their ability to modulate immune responses makes them an even more attractive candidate for regenerative medicine as allogeneic transplant of these cells is feasible without a substantial risk of immune rejection. MSCs secrete various immunomodulatory molecules which provide a regenerative microenvironment for a variety of injured tissues or organ to limit the damage and to increase self-regulated tissue regeneration. Autologous/allogeneic MSCs delivered via the bloodstream augment the titers of MSCs that are drawn to sites of tissue injury and can accelerate the tissue repair process. MSCs are currently being tested for their potential use in cell and gene therapy for a number of human debilitating diseases and genetic disorders. This paper summarizes the current clinical and nonclinical data for the use of MSCs in tissue repair and potential therapeutic role in various diseases.

## 1. Introduction

Stem cells are immature tissue precursor cells which are able to self-renew and differentiate into multiple cell lineages [1, 2]. Mesenchymal stem cells (MSCs), also known as multipotent mesenchymal stromal cells, are self-renewing cells which can be found in almost all postnatal organs and tissues [3, 4]. MSCs have received wider attention because they can be easily isolated from a small aspirate of bone marrow or adipose tissue and can be expanded to clinical scales in *in vitro* condition. Other than these MSCs offer several other advantages like long-term storage without major loss of potency and no adverse reactions to allogeneic MSCs transplant [5]. 

In 1976 Friedenstein et al. firstly described a method for MSCs (referred as “stromal cells”) isolation from whole bone marrow aspirates based on differential adhesion properties. They suggested that these cells are adherent, clonogenic, nonphagocytic, and fibroblastic in nature, with the ability to give rise to colony forming units-fibroblastic (CFU-F) [6]. In late 1980s Owen and Friedenstein reported heterogeneity of the bone marrow stromal cells for the first time [7, 8]. Bone marrow stromal cells were further characterized and named mesenchymal stem cell to describe the subtype of marrow stromal cells involved in the process of mesengenesis [9, 10]. Shortly after these discoveries researchers started to explore the therapeutic application of MSCs [11], since then no adverse effect of MSC transplantation has been reported. In this paper we tried to compile recent advances in the MSCs research and its medical implications.

## 2. Immunophenotype of MSC

The identification of MSCs with the use of specific markers remains elusive. There is no single surface marker, but rather a panel of surface markers which define Human MSCs (hMSCs), derived from fresh tissues or cryopreserved samples. As per the international society for cellular therapy guidelines, MSCs must express CD105 (SH2), CD73 (SH3/4), and CD90 and must be negative for surface markers CD34, CD45, CD14, CD79*α* or CD19, and HLA-DR [9]. hMSCs are also negative for several other antigens like CD4, CD8, CD11a, CD14, CD15, CD16, CD25, CD31, CD33, CD49b, CD49d, CD49f, CD50, CD62E, CD62L, CD62P, CD80, CD86, CD106 (vascular cell adhesion molecule [VCAM]-1), CD117, cadherin V, and glycophorin A. On the other hand, hMSCs are positive for CD10, CD13, CD29 (b1-integrin), CD44, CD49e (a5-integrin), CD54 (intercellular adhesion molecule [ICAM]-1), CD58, CD71, CD146, CD166 (activated leukocyte cell adhesion molecule [ALCAM]), CD271, vimentin, cytokeratin (CK) 8, CK-18, nestin, and von Willebrand factor [5, 12, 13]. Tissue specific expression of surface marker is well noted such as only adipose tissue-derived MSCs express high levels of CD34 [14] and bone-marrow-derived MSCs, but not placenta derived MSCs, express CD271 [15]. Detailed phenotypic expression of surface markers is reviewed elsewhere [16].

## 3. Differentiation Potential of MSC

Other than surface markers MSCs must have ability to adhere to plastic and differentiate into osteoblasts, adipocytes, and chondroblasts under *in vitro* condition [9]. Differentiation is regulated by genetic events, involving transcription factors. Differentiation to a particular phenotype pathway can be controlled by some regulatory genes which can induce progenitor cells' differentiation to a specific lineage. Besides growth factors and induction chemicals, a microenvironment built with biomaterial scaffolds can also provide MSCs with appropriate proliferation and differentiation conditions [17]. Even though MSCs can differentiate into a number of tissues *in vitro*, the resulting cell population does not mimic the targeted tissues entirely in their biochemical and biomechanical properties [18].

### 3.1. Mesoderm Differentiation

Theoretically, mesodermal differentiation is easily attainable for MSCs because they are from same embryonic origin. In the literature also mesoderm (osteogenic, adipogenic, and chondrogenic) differentiation is relatively well studied. A mixture of Dexamethasone (Dex), *β*-glycerophosphate (*β*-GP), and ascorbic acid phosphate (aP) has been widely used for induction in osteogenic differentiation [18, 19]. Osteogenic differentiation of MSCs is a complex process that is tightly controlled by numerous signaling pathways and transcription factors [20]. Runt-related transcription factor 2 (Runx2) and Caveolin-1 are considered a key regulator of osteogenic differentiation which is precisely regulated by numerous activators and repressors [19–21]. Bone morphogenetic proteins (BMPs), especially BMP-2, BMP-6, and BMP-9, have been shown to enhance osteogenic differentiation of MSCs [18]. Smads, p38 and Extracellular signal-Regulated Kinase-1/2 (ERK1/2) are involved in BMP9-induced osteogenic differentiation [22]. At very low concentration BMP-2, vascular endothelial growth factor (VEGF) and basic fibroblast growth factor (bFGF) synergistically promote the osteogenic differentiation of rat bone marrow-derived mesenchymal stem cells. Other than core binding factor alpha-1/osteoblast-specific factor-2 (cbfa1/osf2) [23], Wnt signaling has also been implicated in osteogenic differentiation of MSCs [24]. Recently a study by Alm et al. showed that transient 100 nM dexamethasone treatment reduces inter- and intraindividual variations in osteoblastic differentiation of bone marrow-derived human MSCs [25]. An alternative approach would be to use a scaffold or matrix engineered to provide cues for differentiation. Silicate-substituted calcium phosphate (Si-CaP) supported attachment and proliferation of MSCs was proved to be osteogenesis [26]. In adipogenesis differentiation, Dex and isobutyl-methylxanthine (IBMX) and indomethacin (IM) have been used for induction and have been observed by staining the lipid droplets in cells by Oil Red O solution. Peroxisome proliferator-activated receptors-*γ*2 (PPAR-*γ*2), CCAAT/enhancer binding protein (C/EBP), and retinoic C receptor have been implicated in adipogenesis [17]. Phosphatidylinositol 3-kinase (PI3 K) activated by Epac leads to the activation of protein kinase B (PKB)/cAMP response element-binding protein (CREB) signaling and the upregulation of PPAR*γ* expression, which in turn activate the transcription of adipogenic genes, whereas osteogenesis is driven by Rho/focal adhesion kinase (FAK)/mitogen-activated protein kinase kinase (MEK)/ERK/Runx2 signaling, which can be inhibited by Epac via PI3 K [27].

In chondrogenesis differentiation, transforming growth factor (TGF)-*β*1 and TGF-*β*2 are reported to be involved [28]. Differentiation of MSCs into cartilage is characterized by upregulation of cartilage specific genes, collagen type II, IX, aggrecan, and biosynthesis of collagen and proteoglycans. The emerging results suggested the possible roles of Wnt/**β**-catenin in determining differentiation commitment of mesenchymal cells between osteogenesis and chondrogenesis [19]. A recent report suggested that miR-449a regulates the chondrogenesis of human MSCs through direct targeting of Lymphoid Enhancer-Binding Factor-1 [29]. Elevated **β**-catenin signaling induces Runx2, resulting in osteoblast differentiation, whereas reduced **β**-catenin signaling has the opposite effect on gene expression, inducing chondrogenesis [30]. Fibroblasic Growth factor-2 (FGF-2) can enhance the kinetics of MSC chondrogenesis, leading to early differentiation, possibly by a priming mechanism [31]. 

### 3.2. Ectoderm Differentiation

*In vitro *neuronal differentiation of MSCs can be induced by DMSO, butylated hydroxyanisole (BHA), *β*-mercaptoethanol, KCL, forskolin, and hydrocortisone [17]. Moreover, Notch-1 and protein kinase A (PKA) pathways are found to be involved in neuronal differentiation [32]. In presence of other stimulatory, downregulation of caveolin-1 promotes the neuronal differentiation of MSCs by modulating the Notch signaling pathway [33].

### 3.3. Endoderm Differentiation

In liver differentiation, hepatocyte growth factor and oncostatin M were used for induction to obtain cuboid cells which expressed appropriate markers (*α*-fetoprotein, glucose 6-phosphatase, tyrosine aminotransferase, and CK-18) and albumin production *in vitro* [34]. Recent studies identified methods to develop pancreatic islet *β*-cell differentiation from adult stem cells with desirable results. The resulting cells showed specific morphology, high insulin-1 mRNA content, and synthesis of insulin and nestin [35, 36]. Murine adipose tissue-derived mesenchymal stem cells can also differentiate to endoderm islet cells (expressing Sox17, Foxa2, GATA-4, and CK-19) with high efficiency then to pancreatic endoderm (Pancreatic and duodenal homeobox 1[Pdx-1], Ngn2, Neurogenic differentiation [NeuroD], paired box-4 [PAX4], and Glut-2), and finally to pancreatic hormone-expressing (insulin, glucagon, and somatostatin) cells [37].

## 4. Migration and Homing

The physical niche and migration signals of MSCs provide invaluable information about their role and interactions within the tissue. Bone-marrow-derived MSCs received more attention from researchers in hopes of revealing clues about their therapeutic activity. During *in vivo* condition, it is difficult to locate MSCs' niche. Moreover due to the lack of any specific MSCs marker and difficulties in probing marrow cavities, it is very difficult to track dynamic movement of MSC. Most researchers use genetic markers such as Y-chromosome, when male cells are introduced into females or fluorescent protein reporter genes but these methods do not resolve the dynamics of cellular and temporal responses and are not quantitative [5]. Noninvasive *in vivo *imaging accomplished by using bioluminescence imaging (BLI) can be a possible solution. The main advantage of BLI is that even at very low levels of signal, as few as 100 cells can be detected *in vivo* [38, 39]. Significant advances have been made in this field but still MSCs migration to tissue niche is illusive.

MSCs migration to injured tissues has been reported in radiation-induced multiorgan failure, ischemic brain injury, myocardial infarction, and acute renal failure [40], but the mechanisms that regulate the MSCs migration to the injured tissues are still unknown. Human MSCs express different combinations of the chemokine receptors CCR1, CCR4, CCR7, CCR9, CCR10, CXCR1, CXCR3, CXCR4, CXCR5, and CX3CR1 [41]. The chemokine(s) that control MSCs trafficking are still unknown; while to date, 39 chemokines have been identified with different functions controlling the traffic of hematopoietic cells, in particular leukocytes [41]. Among these chemokines, stromal cell derived factor-1 (SDF-1) is relatively well studied for MSCs migration. 

SDF-1-induced cell migration is mediated by its receptor, CXCR4, which is broadly expressed in cells of the immune system and in the central nervous system (CNS). The role of SDF-1 as an important mediator of stromal progenitor migration to injured tissue has been reported *in vivo *using a rat model of myocardial infarction [42, 43]. Hiasa et al*., *2004 reported that the overexpression of human SDF-1 in the ischemic muscle induced the mobilization of endothelial progenitor cells and improved myocardial healing. Studies also demonstrated that after myocardial infarction the levels of SDF-1 are increased in infarcted tissue and this increase correlates with the number of MSCs that home into the heart [42, 43]. On the other hand, study by Ip et al., 2007 suggested that MSCs use integrin *β*1 and not CXCR4 for their myocardial migration [44]. Moreover, in regenerating skeletal tissues, the MSCs homing may be improved with growth factor delivery, as combined MSCs and erythropoietin infusion gave better results in limb ischemia treatment [45]. Bioactive lipid lysophosphatidic acid (LPA) plays a principal role in the migration of human lung resident MSCs through a signaling pathway involving LPA1-induced beta-catenin activation [46]. Anti-inflammatory environment is more accommodating to the therapeutic hMSCs than a proinflammatory environment [47].

Crossing of the endothelial barrier is another critical step for the tissue migration of circulating cells. Similarly to leukocytes, MSCs adhesion to the endothelial cells represents a critical step and a restricted set of molecules such as selectin-P, integrin *β*1, and VCAM-1 and seems to play critical roles in this interaction [48]. The *in vivo* homing potential of MSCs circulating in the bloodstream to the sites of injury/inflammation can be regulated by adhesion of MSCs to endothelium, achieved by pretreatment of endothelial cells with some proapoptotic agents, angiogenic and inflammatory cytokines, and growth factors, such as interleukin (IL)-8, neurotrophin-3, TGF-*β*, IL-1*β*, TNF-*α*, platelet-derived growth factor, EGF, and SDF-1 [12]. Further studies into understanding the molecular mechanism behind migration and homing will provide an impetus to the use of MSCs for therapeutic purpose. 

## 5. Mechanism of Action/Mode of Action

The mechanism by which MSCs exert their antiproliferative effect have still to be fully elucidated, although several mechanisms and molecules have been proposed that are likely to act in concert and/or in alternate fashion depending on the environment conditions to which MSCs are exposed. Several studies have shown that MSCs are capable of replacing damaged tissues *in vivo *[49, 50]. Multiple tissue engineering approaches have also been reported where undifferentiated or predifferentiated MSCs were delivered with or without help of biomaterial [49, 50]. MSCs have shown promise in replacing various tissues including cartilage, bone, tendon, vasculature, liver kidney, and nerve [51]. However, it remains unclear that how many originally delivered MSCs retain residency in the wounded tissue and maintain the appropriate terminally differentiated phenotype because large amount of transplanted population become apoptotic within the initial phase, or migrate to lungs and liver. Study on stroke and cardiac injury by Li et al. and Askari et al., respectively, suggested that transient MSCs presence appears to be sufficient to elicit a therapeutic effect [52, 53]. Taking together these findings suggests that resident MSCs also work to suppress both transient and perpetual immune surveillance systems and create an ideal healing environment by secreting factors and altering the local microenvironment [51]. 

Since 2002 *in vitro* T-lymphocyte activation and proliferation assays have been used in several studies which resulted in understanding the immunomodulatory effect of MSCs from human, murine, and baboon [54–56]. These studies demonstrated that MSCs were capable of suppressing both lymphocyte proliferation and activation in response to allogeneic antigens. Moreover, MSCs can induce development of CD8^+^ regulatory T (Treg) cells than can in turn successfully suppress allogeneic lymphocyte responses [56] and prohibit differentiation of monocytes and CD34^+^ progenitors into antigen presenting dendritic cells [57]. T cells stimulated in presence of MSCs get arrested in the G1 phase as a result of cyclin D2 downregulation [58]. MSCs are also capable of inhibiting the proliferation of IL-2 or IL-15 stimulated NK cells [59, 60]. MSCs have also been shown to alter B-cell proliferation, activation, IgG secretion, differentiation, antibody production, and chemotactic behaviors [51]. Treatment with *in vitro* expanded allogeneic MSCs successfully resolved severe grade IV acute graft-versus host disease (GvHD) supported *in vivo *immunomodulatory properties of MSCs [61]. Furthermore, MSCs reduce expression of major histocompatibility complex class II (MHCII), CD40, and CD86 on Dendritic cell (DC) following maturation induction [51]. Interestingly, allogeneic MSCs which were differentiated towards a chondrogenic phenotype continued to suppress antigen specific T-cell proliferation in rheumatoid arthritis [62] and genetically engineered MSCs escaped immune rejection and induced ectopic bone formation *in vivo *[56]. However several other reports suggested that the immunomodulatory effects of MSCs are not universal and unconditional and that the MSCs phenotype is transient and context dependent [63].

Cytokine secretion is one of the major therapeutic characteristics of MSCs [64]. MSCs secretion is not limited to factors like TGF-*β*, IL-10, IL-6, cyclooxygenase-1 (COX-1), and COX-2 which are responsible for prostaglandin E2 (PEG2) secretion. MSCs partly inhibited DC differentiation through IL-6 secretion and reduced tissue inflammation by IL-10, TGF-*β*1, and IL-6 secretion [57, 65]. TGF-*β*1 secretion by MSCs suppresses T-lymphocyte proliferation and activation, initiated by IL-1*β* secretion from CD14^+^ monocytes [66]. In fact one study suggested that only the supernatants obtained from cocultures of stromal cells and activated T cells displayed an immunosuppressive effect when added to secondary cultures of proliferating T cells [58, 67]. Taking together MSCs mediated immunosuppression is not exclusively the result of a direct inhibitory effect but involves the recruitment of other regulatory effects. Details about immune-modulation of immune response are reviewed elsewhere [68, 69]. 

## 6. MSCs in Different Diseases

### 6.1. Diabetes Mellitus

Diabetes mellitus (DM) is characterized by hyperglycemia resulting from defects in insulin secretion, insulin action, or both. DM type I or juvenile-onset diabetes is characterized by beta-cell destruction, typically by an autoimmune T cell-mediated mechanism, which usually leads to an absolute deficiency of insulin in the body required for glucose metabolism. Type 2 diabetes or adult onset diabetes is characterized by the inability of insulin to properly metabolize glucose [70]. Despite the different pathogenic mechanisms of Type 1 and Type 2 diabetes, they share common symptoms including glucose intolerance, hyperglycemia and hyperlipidemia. DM is also implicated in the other pathologies such as adult blindness, kidney failure, amputation of leg and feet, pregnancy complications, and heart attack [70]. Current insulin therapy is neither capable of completely mimicking endogenously secreted insulin released nor is safe as it often causes hypoglycemic coma [70]. Thus, strategies to promote either the expansion of existing beta-cells within the body or the supply of stem cell derived insulin-producing cells would provide future treatment options. As previously discussed MSCs are able to differentiate into several cell types making them a potentially important source for the treatment of debilitating human diseases such as diabetes [71]. 

*In vitro* differentiation of MSCs in insulin-producing cells (IPCs) is well documented. The differentiation of bone marrow-derived MSCs is achieved by multistep differentiation protocols. The protocols include a combination of nicotinamide, activin A, and *β*-cellulin in high glucose medium. At the end of the culture, differentiated cells show a similar morphology to that of pancreatic islet-like cells-high PDX-1, insulin and glucagon genes expression, and glucose dependent insulin production [72]. Similar results were also reported when umbilical cord blood MSCs were used as a source of IPCs. Obtained islet like clusters released insulin and C peptide in response to physiological glucose concentration *in vitro *[73]. Generation of new islet from pancreatic epithelial cells *in vitro *is also reported [74]. These *in vitro* islets contained alpha and delta cells, which responded well to *in vitro* glucose challenge and once implanted in nonobese diabetic (NOD) mice reversed insulin-dependent diabetes [74]. Combined transfection of the three transcriptional factors, PDX-1, NeuroD1, and MafA, causes differentiation of bone marrow MSCs into insulin-producing cells [75]. In another study, bone-marrow-derived MSCs were converted *in vitro* into insulin-producing cells by suppressing two genes, repressor element-1 silencing transcription factor/neuronal restrictive silencing factor (Rest/Nrsf) and sonic hedgehog (Shh) and by overexpressing Pdx1. The reprogrammed bone-marrow-derived MSCs expressed both genes and proteins specific for islet cells [76]. Although it is very preliminary, most promising results for the cell based therapy for diabetes were reported by Timper et al. when they showed the possibility to generate IPCs from adipose derived MSCs [77]. 

The immunomodulatory capability of MSCs is considered equally important for diabetes treatment, especially in diabetes type I. Preclinical study by Ezquer et al. suggested that antidiabetic effect of mesenchymal stem cells is unrelated to their transdifferentiation potential but to their capability to modulate immune response and to modify the pancreatic microenvironment [78]. They suggested that in the pancreas of mice with DM Type-I treated with MSCs, a cytokine profile shift from proinflammatory to antinflammatory was observed. MSC transplantation did not reduce pancreatic cell apoptosis but recovered local expression and increased the circulating levels of epidermal growth factor, a pancreatic trophic factor [78]. On the other hand, a study by Ho et al. suggested that lasting therapeutic effect of MSCs was due to MSC engraftment and differentiation in insulin producing cells and also due to immunomodulation properties [79]. Although the mechanism of action was not defined, in phase I clinical trial Wharton's jelly derived MSCs show long-term beneficial effect on newly diagnosed DM Type-I patients. Compared to DM Type-I, limited study has been done on MSCs transplantation in DM type 2 (T2D), but initial preclinical and pilot clinical studies showed encouraging results. MSC inoculums improved metabolic control in experimental models of T2D [80–83]. Usage of MSCs was also implemented in several diabetes related complications like cardiomyopathy, nephropathy, polyneuropathy and diabetic wounds [71]. Chronic hyperglycemia is responsible for myocardial remodeling and is a central feature in the progression of diabetic cardiomyopathy (DCM) which is characterized by hypertrophy and apoptosis of cardiomyocytes and alterations in the quality and composition of the extracellular matrix (ECM) resulting in increased collagen deposition [71]. Matrix Metalloproteinase (MMP) 2 and 9 activities play central role in the pathology of cardiomyopathy; decreased MMP-2 activity leads to increased collagen accumulation and increased MMP-9 activity leads to increased apoptosis of endothelial cells, reduction of capillary density, and poor myocardial perfusion [84, 85]. In rat model of DCM, intravenous administration of bone-marrow-derived MSCs improved myogenesis and angiogenesis [86]. In this study MSCs transplantation increases in MMP-2 activity and decreases in MMP-9 activity which in turn increases myocardial arteriolar density and decreases collagen volume resulting in attenuation of cardiac remodeling and improved myocardial function [86]. In a mice model systematic administration of MSCs showed improvement of kidney function and regeneration of glomerular structure as MSCs are able to reconstitute necrotic segment of diabetic kidneys [87, 88]. As MSCs are not able to proliferate in kidney [89], an alternative scenario for improvement of kidney function could be the ability of MSCs to scavenge cytotoxic molecules or to promote neovascularization [71]. Diabetic polyneuropathy (DPN) is the most common complication of DM which is characterized by damage to nerve fibers. The central features of DPN are neural cell degeneration and decreased nerve blood flow (NBF). One month after the intramuscular injection MSCs found to be producing bFGF and VEGF which led to increase in the ration capillaries to muscle fibers followed by improvement of hyperalgesia and a corresponding functional improvement of neural fiber [90]. Although studies suggested that MSCs have the capacity to differentiate into neural cells *in vitro*, this was not observed during *in vivo *studies on diabetic rat model [90]. Studies on rat and mice showed that systematic and local administration of bone marrow-derived MSCs improves healing of diabetic wounds. MSCs injection resulted in increase in several growth factors important for successful wound healing. These factors stimulated cell adhesion at the site of injury and induced cell to secrete more chemokines resulting in neovascularization and formation of inflammation infiltrate, containing predominantly mononuclear cells, without tissue necrosis [91]. Promising preliminary and preclinical studies have led to phase I and phase II clinical trials, the results of which are awaited. Outcome of these studies will decide the future of cell-based therapy for the most devastating degenerative disease of mankind. 

### 6.2. Cardiac Diseases

Ischemic heart disease is the leading cause of death in developed countries and has significant morbidity rate. MSCs' application in heart repair is well studied in preclinical and clinical studies. After an acute myocardial infarction (MI), the heart has limited capacity for self-renewal properties and undergoes remodeling with resulting depressed left ventricular function [92]. In the last decade intense investigations have been done on MSCs as a future cell-based therapeutic strategy for cardiac repair and many of these studies have been translated into clinical trials [92]. The various possible mechanisms of MSC mediated cardiac improvement have been suggested which, includes somatic reprogramming, transdifferentiation, paracrine signaling, and direct electrophysiological coupling [93]. Numerous* in vivo *rodent and swine studies have demonstrated the ability of MSCs to engraft and differentiate within the heart. 

Studies by Shake et al. and Toma et al. successfully demonstrated that injected MSCs engrafted into scarred myocardium and expressed cardiomyocyte markers like *α*-actin, desmin, tropomyosin, and myosin heavy chain [94, 95]. In swine model of chronic ischemic cardiomyopathy, Quevedo et al. reported the capacity of allogeneic MSCs to engraft and differentiate into cardiomyocytes, smooth muscle cells, and endothelium [96]. Several other studies also reported that MSCs differentiate into cardiomyocytes *in vivo *[97–99]. In contrast to reports of engraftment Dixon et al. showed that male mesenchymal precursor cells transplanted into post-MI sheep were unable to demonstrate engraftment [100]. Functional recovery after MSCs transplantation is well documented and well accepted. MSCs transplantation in most animal models of MI generally resulted in reduced infarct size, improved left ventricular ejection fraction (LVEF), and increased vascular density and myocardial perfusion. On the basis of rigorous preclinical studies and demonstrated safety aspect, clinical trials have been initiated for MI and ischemic cardiomyopathy. Intracoronary infusion of bone-marrow-derived MSCs in subacute MI showed improvement in perfusion defects at 3 months after the therapy and left ventriculography demonstrated improved ejection fraction (EF) and left ventricular chamber size [101]. Similarly, intravenous injection of MSCs in acute MI demonstrated a reduction in ventricular arrhythmias and improved pulmonary function where the patients had a 6% increase in EF at 3 months [102]. In ischemic cardiomyopathy transendocardial intramyocardial injection of MSCs demonstrated reverse remodeling and improved regional contractility of treated scar, 3 months after injection which persisted for 12 months. The improvement was also reflected in end-diastolic volume (EDV) and end-systolic volume (ESV) [103]. To improve the engraftment of transplanted MSCs a small scale clinical trial was performed on patients with chronic MI who were treated with a collagen scaffold previously seeded with bone marrow mononuclear cells but only marginal ventricular wall remodeling and an improved diastolic function were detected [104, 105]. 

Despite numerous studies on the transplantation of MSCs in patient and animal models, insight into the mechanistic issues underlying the effect of MSC transplantation remains vague. A recent study suggested the importance of IL-6 secretion and activation of Janus kinase/signal transducers and activators of transcription (JAK-STAT) in cardiac repair by transplanted MSCs [106]. A recent study demonstrated that paracrine signaling resulted in increased survival of ventricular myocytes by Akt induced change in calcium signaling which resulted in antiapoptotic effect by transplanted MSCs [107]. The frequency of MSCs engraftment and differentiation in the heart is low compared with the robust functional recovery observed after cell transplantation which suggests that engraftment and differentiation might not be the predominant effect. As mentioned before MSCs are known to secrete soluble paracrine factors that have been postulated to contribute to endogenous cardiomyogenesis and angiogenesis. However, the mechanism through which these factors act is yet to be explored. 

### 6.3. Liver Diseases

Liver transplant is the most preferred solution in case of liver diseases but donor organ shortage is the main reason why whole organ or hepatocyte transplants cannot be done frequently. Therefore, generation of hepatocyte-like cells from MSCs has become a real alternative to the isolation of primary hepatocytes. Under specific growth conditions, MSCs have been shown to adopt functional features of differentiated hepatocytes and successfully engrafted into mouse liver [108]. In allylalcohol- (AA-) treated rat liver, xenografting of allogeneic MSCs differentiated into hepatocytes-like cells which showed positive immunostaining for albumin, CK-19, CK-18, and asialoglycoprotein receptor [109]. MSCs facilitate recovery from chemically induced liver damage and also help in decreasing liver fibrosis in rat model [110]. Similar result was observed in rat model of liver cirrhosis [111]. Injected MSCs were diffusely engrafted in the liver parenchyma and showed CK-19 positive and albumin producing hepatocytes. Although the engraftment rate was low, MSCs showed therapeutic effects including repair of damaged hepatocytes, intracellular glycogen restoration, and resolution of fibrosis. Similarly, bone marrow-derived MSCs showed protection against experimental liver fibrosis in rats model [112–114]. Both MSCs and MSCs derived hepatocytes, transplanted by either intrasplenic or intravenous route, engrafted in mice liver, differentiated into functional hepatocytes, and rescued liver failure [115]. In contradictory to this finding, Burra et al. suggested that systemic administration of umbilical cord MSCs accelerates the resolution of an acute liver injury without any differentiation and manipulation [116]. MSCs transplantation not only showed improvement in liver function caused by degenerative disease but also showed significant improvement in liver damage caused by Schistosoma japonicum. In combination with conventional drug praziquantel, MSCs transplantation prolonged the survival time of infected mice by reducing egg granuloma diameter and decreasing the concentrations of serum TGF-*β*1 and hyaluronic acid [117]. Cytoprotective mechanism of MSCs is still very illusive. Recently, a study suggested that the cytoprotective due to the promotion of antioxidant response by bone marrow-derived MSCs [118]. A recent study suggested that MSCs are recruited to injured liver in a beta1-integrin and CD44 dependent manner [119]. In preclinical studies researcher observed that mode of stem cell transplantation affects the outcome. In swine model of acute liver failure, transplantation by portal vein gave best result and not only supported liver regeneration but also prolonged the host survival [120]. On the other hand, in rat liver fibrosis model, transplantation through intravenous injection has been shown to give best results and protects the liver against fibrosis through IL-10 expression [121]. 

The potential of MSCs in liver repair is also studied in humans. Phase I and II clinical trial for liver cirrhosis suggested that both differentiated [115] and undifferentiated MSCs transplantation improved liver function [122–124]. Followup of patients at 3 and 6months transplant revealed partial improvement of liver function tests with elevation of prothrombin concentration and serum albumin levels, decline of elevated bilirubin and Model for End-Stage Liver Disease score (MELD) [122]. In decompensated liver cirrhosis, umbilical cord-MSCs transplantation showed a significant reduction in the volume of ascites. Umbilical cord -MSC therapy also significantly improved liver function, as indicated by the increase of serum albumin levels, decrease in total serum bilirubin levels, and decrease in the MELD scores during one-year follow-up studies [125]. Another elaborated clinical observation for liver failure suggested that autologous bone-marrow MSCs transplanted patients showed marked improvement in the level of alanine aminotransferase, albumin, total bilirubin, prothrombin time, and MELD from 2-3 weeks after transplantation but long-term followup did not show any significant difference between control and transplanted group [126]. 

Although preclinical and clinical studies have given promising results, thorough investigations are required to translate these studies in routine treatment. Scientists are also looking forward to improve therapeutic effect of MSCs by applying pretreatment with different chemical [127] and testing genetically modified MSCs [128]. 

### 6.4. Kidney Diseases

There are enough reports of MSCs repopulating the damaged kidney with varying degrees of significance. Intraparenchymal injection of bone-marrow-derived MSCs reduces kidney fibrosis after ischemia-reperfusion in cyclosporine-immunosuppressed rats [129]. Initial experimental studies reported that the exogenous administration of MSCs to mice with acute renal injury could promote both structural and functional renal repair via the transdifferentiation of MSCs into tubular epithelium [130]. However, only 2–2.5% of the injected MSCs showed engraftment [130, 131]. MSCs by virtue of their tropism for damaged kidney and ability to provide a local prosurvival environment may represent a useful strategy to preserve podocyte viability and reduce glomerular inflammation and sclerosis [132]. Furthermore, a study with female mice which received a male bone marrow for tubular injury showed about 4% of tubular cells to be positive for Y-chromosome which suggested that a small but significant amount of engrafted bone-marrow-derived cells participated in kidney regeneration [133]. In contradictory to this, other study reported that arterial injection of MSCs reduced the necrosis, improved kidney function, and increased the proliferation of mesangial cells and their expression of *α*-smooth muscle actin (*α*-SMA), yet no incorporation of MSCs in to kidney structures was seen [134]. These reports demonstrate that the direct engraftment of exogenously administered, and transdifferentiating MSCs is not the predominant mechanism in which MSCs enhance renal repair [134]. There is increasing evidence that MSCs can elicit kidney repair through paracrine and/or endocrine mechanisms, where they release trophic growth factors that modulate the immune response and consequently mediate repair [134]. The ability of MSCs to inhibit the release of pro-inflammatory cytokines and secrete a variety of trophic growth factors that promote angiogenesis, mitogenesis, and proliferation whilst reducing apoptosis may collectively mediate the protective and regenerative effects in the kidney of laboratory rodents [134, 135]. A recent study of targeted delivery of bone-marrow-derived MSCs challenged this belief. In their study researcher not only showed homing of bone-marrow-derived MSCs but also showed the recovery of kidney in rat model of acute kidney injury [136]. In a pilot clinical study of chronic kidney disease, two intravenous transplantation of ~1 million MSCs/kg body showed significant difference between each of serum creatinine and creatinine clearance levels before and after MSC injection at 1, 3, and 6 months after infusion [137].

### 6.5. Bone Diseases

Because of the lack of an adequate supply of autologous bone grafts and the unsuitability of allografts, there has been some impetus to use MSCs to encourage repair. Studies on murine model showed very promising results especially for bone repair and metabolic bone disorders [138]. Since their first use in 1951, MSCs have been successfully applied for bone regeneration [138]. Study on the femur of athymic rats showed that a ceramic scaffold loaded with expanded MSCs gave significantly increased bone formation compared to control group [139]. 

*In vitro* expanded MSCs loaded on porous hydroxyapatite scaffolds were used to cure bone nonunion and diaphyseal defects which resulted in good integration of implant [140, 141]. Angiographic evaluation of implants after seven years showed vascularisation of the grafted zone, which is believed to be vital for the survival and future stability of the graft. Study by another research group showed that differentiated bone-marrow-derived stem cells can help patient to obtain the target length of femora and tibiae in patients undergoing distraction osteogenesis [142]. MSCs have been successfully used in the treatment of steroid-induced osteonecrosis of the femoral head [143]. Scaffolds seeded with bone-marrow-derived MSCs have also been used in spinal fusion but further investigation using proper controls is necessary before we make any final conclusions [144]. SDF-1 and its receptor CXCR4 have been shown to act as a potential homing signal for MSCs in bone healing [145]. Another study showed that bone healing depended on the number and concentration of transplanted autologous MSCs, which suggested at least 1000 or more MSCs per cm^3^ are required to achieve union [146]. The combination of mesenchymal stem cells, platelet rich plasma, and synthetic bone substitute was found to be more effective in inducing new bone formation (osteogenesis) than the use of platelet rich plasma combined with synthetic bone substitute and the use of synthetic bone substitute alone  [145].

Osteogenesis imperfecta (OI) is a connective tissue disorder characterized by bone fragility and other evidence of connective tissue malfunction. When MSCs from wild-type mice were infused into transgenic mice that had a phenotype of fragile bones resembling OI, the MSCs served as a source for continual renewal of cells in a number of nonhematopoietic tissues [147]. Adult bone marrow donor cells from transgenic mice engrafted into hematopoietic and nonhematopoietic tissues and synthesized up to 20% of all type I collagen in the host bone and also eliminated the perinatal lethality of mice with dominant OI [148]. Allogeneic bone marrow transplant in 3 children with OI showed osteoblast engraftment, which was nearly 2.0% donor cells, resulted in histologic changes indicative of new bone formation and increased in total body bone mineral content [149]. However, study had only 6 months of clinical followup and did not directly compare results with controls. Same group showed linear growth, total body bone mineral content, and fracture rate in 3 children (out of 5) with severe OI [150]. With increasing time after transplant, growth rates slowed and eventually plateaued, whereas bone mineral content continued to increase [150]. In another study Horwitz et al. treated six children suffering from OI by systemic infusion of MSCs for bone regeneration. Five children showed acceleration of bone growth compared with matched unaffected children [151]. However, direct application of MSCs to the fracture is deemed to be more practical [138]. Le Blanc et al. transplanted allogeneic HLA-mismatched MSCs in a 32 weeks old fetus and proved the participation of transplanted cells in bone turnover using Y-chromosome specific probe [152]. Allogeneic transplant of patient's osteoblasts proved to be helpful in hypophosphatasia, a heritable metabolic disorder [153]. Results showed that patient osteoblasts were replaced with donor cells and marked improvement was observed in the bone without any changes in biochemical feature of hypophosphatasia which was confirmed clinically and radiologically [153]. 

### 6.6. Autoimmune Diseases

The property of MSCs to modulate the functions of several immune effector cells could be involved in pathogenesis of autoimmune diseases which makes them a useful tool for treatment of autoimmune diseases [154]. One of the priority target disease is GvHD which is otherwise untreatable and fatal. The first report on successful use of MSCs for treatment of severe steroid-refractory acute GvHD was in 2004, where ex vivo expanded haplo-identical human MSCs were used [61]. In phase I and phase II clinical trial, MSCs from haplo-identical donors were given in 18 cases and 69 cases were given MSCs from HLA mismatched donors. The results showed complete response in 30 patients and no improvement in 9 patients out of the 55 steroid resistant severe acute GvHD patients [155]. Of the 31 acute GvHD patients treated in another phase II clinical trial using allogeneic MSCs, 94% showed initial response to MSCs, 77% showed complete response, and 17% showed a partial response with no infusion related toxicity or ectopic tissue formation [156]. Interestingly, a large scale phase III clinical trial including 192 acute GvHD patients and 260 steroid-resistant GvHD patients reported in 2009 by Mills et al. showed mixed results. The results of this study indicated that the success of MSC treatment may depend on the type of tissue affected by GvHD patients (http://www.clinicaltrials.gov/, NCT00366145). 

Therapeutic benefits of MSCs have also been hinted for Crohn's disease [157]; however, large scale clinical studies are required to obtain concrete results. A role of MSCs in Crohn's disease is recently reviewed by Dalal et al. [158] and not discussed in detail in this review. Experimental autoimmune encephalomyelitis (EAE) is an autoimmune disease of the CNS which involves T cells and macrophages. Currently, the established treatment for EAE is based on targeting T cells to induce immunosuppression or tolerance. Moreover, many studies have confirmed the therapeutic potential of human and mouse MSCs for EAE treatment by demonstrating improved clinical progress, stimulation for tissue repair, decrease in demyelination, and infiltration of CNS by T cells and macrophages [159–164]. Though there is limited evidence for engraftment of MSCs in CNS for a prolonged period of time [165, 166], MSCs in periphery appear to support tissue repair and stop autoimmune disruption in CNS. 

In a study by Yamout et al, 10 patients with advanced Multiple Sclerosis (MS) were treated with ex vivo expanded bone-marrow-derived MSCs, 50% of which showed beneficial results, suggesting that MSCs are safe and feasible for use in the treatment of MS patients [167, 168]. Amylotrophic lateral sclerosis (ALS) is an autoimmune disease which occurs due to loss of upper and lower motor neurons in the cerebral cortex, brainstem, and spinal cord which leads to death within five years after first appearance of symptoms [169]. In a phase I/II study patients with MS and ALS were treated with intravenous MSC infusion which leads to increase in the proportion of CD4^+^ CD45^+^  T_reg_ cells in the peripheral blood of the patient [170]. Despite promising preclinical results, clear evidence of the beneficial effect of MSCs for the treatment of neurodegenerative disorders is lacking [171, 172]. Furthermore, human clonal MSCs have been reported in recovering pancreatic function in rat models with mild and severe acute pancreatitis (AP) by preventing T cell infiltration, decreasing the expression of inflammatory mediators or cytokines, and by stimulating Foxp3 regulatory T cells [64]. Despite encouraging phase 1/2 studies, no positive data on autoimmune diseases (except GvHD) from randomized clinical studies are as yet available in peer-reviewed journals. 

## 7. Future Direction

The data available till date does not clearly support differentiation and engraftment but anonymously supports its immunomodulating properties. So far we know the molecules that MSCs use for modulating immune effector cells, some of which could also be involved in pathogenesis of autoimmune diseases. However, the underlying mechanisms through which these molecules act are still unclear. Moreover, MSCs are not only able to immunomodulate the immune cells, but also can escape immune rejection. This property however, is dependent on the microenvironment surrounding the MSCs. Recent reports suggest that the inflammatory environment associated with autoimmune diseases might alter the MSC polarization towards immunosuppressive or immunostimulating phenotype [63]. Interestingly, there are few reports where MSCs have protected from a disease in one case and worsened the clinical parameters in the other with the same disease [173, 174]. For such contradiction, it can be argued that the difference in the time parameter when the MSCs were infused after disease induction may lead to diverse inflammatory environments surrounding MSCs in both cases which in turn can influence the function of MSCs. Therefore, scrutinizing the patient's microenvironment before the treatment can help in deciding how the patient will respond to MSC therapy for a particular disease. Such an understanding can have a profound impact on the use of MSCs in clinical setting. Therefore, further studies should be aimed towards comprehending the mechanisms underlying immunomodulation by MSCs to be able to use MSCs for therapeutic purpose.

The clinical uses of MSCs are not limited to treatment of autoimmune diseases; MSCs have also been tested for use in tissue regeneration, as cell vehicles for gene therapy and enhancement of hematopoietic stem cell engraftment. Additionally, more recently a concept of engineered MSCs has been proposed for cancer treatment [175–178]. The use of MSCs as an isolated treatment of cancer is debatable; however, a number of intelligent studies have successfully demonstrated the use of MSCs engineered with specific antitumor genes in targeting cancerous cells and thereby reducing tumor progression [179–183]. These anticancer genes containing MSCs are capable of localizing to a specific tumor site irrespective of tumor type or invasiveness and deliver the anticancer agents [175–178]. Hence well-planned further studies are required to apply this concept in actual clinical setting outside the laboratory.

Fortunately, the clinical studies reported so far have not suggested any critically adverse effects of MSCs on a disease condition in significant number of cases. Therefore, use of MSCs in therapeutics can be considered as safe. However, more data supporting long-term safety, immunogenicity of MSCs in nonimmunocompromised animals, suitable source and number of cells to be infused, is required [64]. Although currently there is lack of consistency in certain areas of MSC therapeutics, the potential of immunomodulatory properties of MSCs is remarkable in order to form the basis of future therapeutics.
